# Hematological Abnormalities of Pulmonary Tuberculosis Patients with and without HIV at the University of Gondar Hospital, Northwest Ethiopia: A Comparative Cross-Sectional Study

**DOI:** 10.1155/2018/5740951

**Published:** 2018-12-30

**Authors:** Feven Abay, Aregawi Yalew, Agumas Shibabaw, Bamlaku Enawgaw

**Affiliations:** ^1^Department of Physiology, School of Medicine, College of Medicine and Health Sciences, University of Gondar, Gondar, Ethiopia; ^2^Department of Hematology and Immunohematology, School of Biomedical and Laboratory Sciences, College of Medicine and Health Sciences, University of Gondar, Gondar, Ethiopia; ^3^Department of Medical Laboratory Sciences, College of Medicine and Health Sciences, Wollo University, Dessie, Ethiopia

## Abstract

**Background:**

Hematological abnormalities are common in pulmonary tuberculosis (PTB) patients, which is one of the major public health problems worldwide. However, there is paucity of information about the hematological profile of PTB patients with and without HIV in the study area. Therefore, this study aimed to assess hematological abnormalities of pulmonary tuberculosis patients with and without HIV at the University of Gondar Hospital, Northwest Ethiopia.

**Methods:**

A comparative cross-sectional study was conducted at the University of Gondar Hospital. Sociodemographic data was collected using a pretested, structured questionnaire. Five milliliters of venous blood sample was collected and divided into a 3 ml EDTA tube for complete blood count with the Cell Dyn 1800 hematological analyzer and a 2 ml citrated tube for erythrocyte sedimentation rate determination. Data were entered into Epi Info version 3.5.3 and then transferred to SPSS 20 for analysis. The independent samples* t*-test was used to compare the mean values of hematological parameters between PTB patients and PTB-HIV coinfected patients.

**Result:**

A total of 100 study subjects (50 PTB and 50 PTB-HIV coinfected) were included with a mean age of 31.3 ± 10.3 years for PTB patients and 32.1 ± 9.2 years for PTB-HIV coinfected patients. In this study, there were significantly lower mean values of Hgb (*P* = 0.049), platelet count (*P* < 0.001), and neutrophils counts (*P* = 0.007) among PTB-HIV coinfected patients when compared with PTB patients. Of the PTB infected patients 46% were anemic, 6% leukopenic, 22% neutropenic, 8% lymphopenic, and 8% thrombocytopenic. On the other hand, of the PTB-HIV coinfected patients 60% were anemic, 14% leukopenic, 66% neutropenic, 12% lymphopenic, and 20% thrombocytopenic. ESR value was increased in all patients.

**Conclusion:**

This study demonstrated high prevalence of neutropenia, anemia, and thrombocytopenia among PTB-HIV coinfected patients. HIV coinfection worsens hematological abnormalities of PTB patients. Assessment of hematological parameters can be used as an indicator in the diagnosis and follow-up of PTB patients coinfected with HIV. We recommended assessment of PTB patients with or without HIV for various hematological disorders such as neutropenia, anemia, and thrombocytopenia.

## 1. Introduction

Tuberculosis (TB) is the ninth leading cause of death worldwide and the leading cause from a single infectious agent, ranking above HIV/AIDS, and the leading killer among HIV-positive people. TB had a global incidence of 10.4 million and mortality of 1.3 million among HIV-negative people and an additional 0.4 million deaths among HIV-positive people in 2016 [1]. Ethiopia is one of the 30 high TB and TB-HIV burden countries in the world [1]. One study in Ethiopia indicates about 27.7% TB-HIV coinfection in the Amhara region, Ethiopia [2]. The bacterium responsible for TB,* Mycobacterium tuberculosis*, can affect different organs in the human body. The demonstrable changes that PTB plus HIV, PTB, and HIV seropositive patients have in their hematological and metabolic values showed varied pictures as a result of the phase of the infection and the causative agents,* M. tuberculosis *and HIV [3].

The hematopoietic system is seriously affected during TB infection. Both myeloid and lymphoid cell lines and plasma components are affected [4]. Specifically, in pulmonary tuberculosis many hematological abnormalities are common and they are valuable aids to the diagnosis [5]. These abnormalities are useful indicators providing a clue to diagnosis, assessing the prognosis, and indicating the complication of underlying infection and response to therapy [6].

Although anemia is the most common complication of both TB and HIV infections, leukocytosis, thrombocytosis, monocytosis, and lymphocytosis are the frequent reported abnormalities [4, 5, 7, 8]. This suggests a systemic response of a patient to active inflammatory responses [9, 10]. HIV associated hematological abnormalities are dependent on the level of virus replication, and severe abnormalities are observed in the late stage of AIDS with high viremia. This is worsened when there is TB coinfection where all blood cell lineages are involved [4, 11]. These often pose a great challenge in the comprehensive management such as cytopenias, anemia, and neutropenia which are caused by inadequate production of cells because of suppression of the bone marrow by the HIV infection through abnormal cytokine expression and alteration of bone marrow microenvironment [12, 13].

There are limited studies conducted on the hematological profiles of pulmonary tuberculosis patients with and without HIV/AIDS in developing countries, especially in Ethiopia. To the best of our knowledge, there is scarcity of data in Ethiopia that assess the hematological profile of PTB patients with and without HIV in order to diagnose changes in hematological parameters and monitor treatment outcomes of PTB patients. Hence, this study was designed to determine the magnitude of hematological abnormalities among PTB patients at the University of Gondar Hospital.

## 2. Methods

### 2.1. Study Setting, Design, and Population

An institution based comparative cross-sectional study was conducted at the University of Gondar Hospital which is located at Gondar, 740 km far from Addis Ababa. The University of Gondar Hospital is a tertiary teaching hospital that serves more than 5 million people in and around Gondar town. The hospital has a TB clinic for the treatment and follow-up of patients which is operated by the National Tuberculosis and Leprosy Control Program (NTLCP) of Ethiopia. According to the rules of thumb that have been recommended by VanVoorhis and Morgan, 30 participants per group are required to detect real differences, which could lead to about 80% power [14]. Thus, a total of 100 pulmonary tuberculosis adult patients (50 PTB and 50 PTB-HIV coinfected individuals) whose sputum was confirmed three times for AFB by the Ziehl-Neelsen method with and without HIV were selected conveniently. The study participants were categorized as those who had only PTB and PTB-HIV coinfected individuals. PTB patients on anti-TB drugs, TB-HIV patients on highly active antiretroviral therapy, patients who had renal or liver failure, pregnant women, and those patients who were on myeloid-suppressive drugs were excluded from the study.

### 2.2. Data Collection and Laboratory Analysis

All sociodemographic and clinical data were collected using pretested, structured questionnaires. Diagnosis of pulmonary TB was done by examination of three sputum smears by the Ziehl-Neelsen staining method for acid fast bacilli (AFB). Chest radiographs and pathological investigations were also used to support the diagnosis. Then about 3 ml of venous blood was collected with a K_3_EDTA anticoagulant tube from each study participant by the experienced laboratory technologist. After the blood was collected it was analyzed by the Cell Dyn 1800 (Abbott Laboratories, Abbott Park, Illinois, USA) analyzer for the determination of hematological parameters. Additionally, erythrocyte sedimentation rate (ESR) was measured by using the Westergren method. To ensure quality of data, hematological quality control materials were analyzed parallel to the patients' samples and manual differential and blood film examination were performed for any suspected flags in the analyzer.

### 2.3. Outcome Definitions

Anemia was defined as Hgb< 13 g/dl for males and Hgb <12 g/dl for females. Hemoglobin values of 9.0–11.0 g/dl for women and 9.0–12.0 g/dl for men were considered as mild while Hgb values 8.0–9.9 gm/dl and Hgb < 8.0 g/dl were considered as moderate and severe anemia, respectively [15]. On the other hand, leucopenia, neutropenia, lymphopenia, and thrombocytopenia were defined as WBC < 3.2 × 10^3^ cells/*μ*l, 1.6 × 10^3^ cells/*μ*l, 1.0 × 10^3^ cells/*μ*l, and 128 × 10^3^ cells/*μ*l, respectively [16].

### 2.4. Statistical Analysis

Data were checked for completeness, cleaned, and entered into Epi Info version 3.3.5 and then transferred to SPSS version 20 for analysis. Frequencies and cross tabulations were used to summarize descriptive variables. The mean, median, and standard deviation were determined for each hematological parameter and presented with tables and graphs. To determine the mean difference in the hematological profiles between PTB patients and PTB-HIV coinfected patients, the independent* t*-test was used. The results were presented with tables and graphs. A* P* value less than 0.05 was considered as statistically significant.

## 3. Ethical Considerations

Ethical approval was obtained from the School of Biomedical and Laboratory Sciences, Research and Ethical Review Committee, University of Gondar. All study participants were informed about the aim of the study and its procedure and written informed consent was obtained. Confidentiality was kept and participation was voluntary. The patients' in-clinic evaluations were managed following the routine patients' management system.

## 4. Results

### 4.1. Sociodemographic Characteristics of the Study Participants

A total of 50 pulmonary tuberculosis (PTB) and 50 PTB-HIV coinfected patients participated in this study with a male-to-female ratio of 1:1. The mean age was 31.3 ± 10.3 years for PTB patients and 32.1 ± 9.2 years for PTB-HIV coinfected patients (Table 1).

### 4.2. Hematological Profile of PTB and PTB-HIV Study Participants

In this study the mean values of hematological parameters among PTB patients with and without HIV were determined and presented in Table 2. Based on the analysis, the mean value of total WBC count was 7.3 ± 3.1 x 10^3^ cells/*μ*l for PTB patients and 7.0 ± 3.2 x 10^3^ cells/*μ*l for PTB patients with HIV, RBC count was 4.6 ± 0.85 × 10^6^ cells/*μ*l for PTB patients and 4.12 ± 0.72 x 10^6^ cells/*μ*l for PTB patients with HIV, and platelet and ESR observed values were 336.4 ± 152.3 x 10^3^ cells/*μ*l and 65.3 ± 35.7 mm/hr among PTB patients and 256.9 ± 131.7 × 10^3^ cells/*μ*l and 66.7 ± 28.6 mm/hr among PTB-HIV coinfection patients, respectively. Independent* t*-test analysis showed that Hgb, platelet, and neutrophils count values were significantly different among PTB and PTB-HIV patients (*P* < 0.05). However, there was no statistically significant difference in WBC, RBC, HCT, MCV, MCH, MCHC, lymphocyte, mixed cell, and ESR values between PTB and PTB-HIV patients.

### 4.3. Hematological Disorders

**Figure 1:**
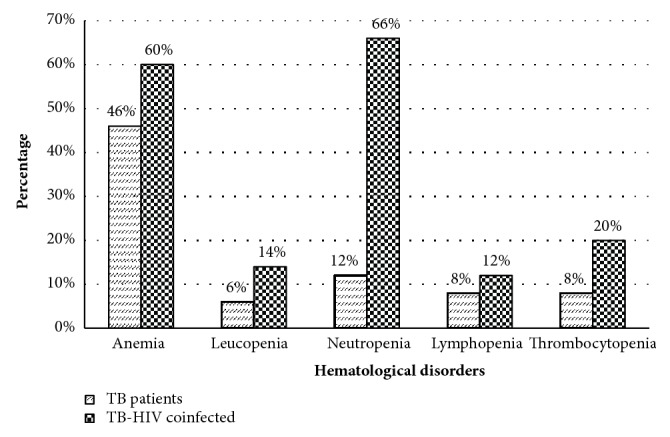
Hematological abnormalities among PTB patients and PTB-HIV coinfected patients at the University of Gondar Hospital, Northwest Ethiopia.

About 46% of the PTB patients had developed anemia. Of anemic patients, 47.48%, 47.8%, and 8.69% had mild, moderate, and severe anemia, respectively. Among PTB-HIV study participants the overall prevalence of anemia was 60%. Of these 40%, 56.7%, and 3.33% were with mild, moderate, and severe anemia. The prevalence of leucopenia, neutropenia, and lymphopenia was 6%, 22%, and 8% among PTB patients, while it was 14%, 66%, and 12% among PBT-HIV coinfected patients, respectively. Low platelet count was observed among 8% of PTB patients and 20% of PTB-HIV coinfected patients (Figure 1).

## 5. Discussion

In this study 46% of the PTB and 60% of the PTB-HIV coinfected patients had developed anemia with the majority of them having a moderate type of anemia followed by mild anemia. In this study, prevalence of anemia is higher compared to a study in Korea (31.9%) [17] but lower than the prevalence of 73% in Iran [18] and 74% in India [11]. The high prevalence of anemia is supported by several studies which showed that high prevalence of anemia among pulmonary TB patients with and without HIV coinfection. Various pathogeneses have been suggested in TB associated anemia, but most studies have showed infection of the hematopoietic progenitor cells, effect of treatment on erythropoiesis and folate activity, nutritional deficiencies and malabsorption, absence or depletion of bone marrow iron, and suppression of erythropoiesis by inflammatory mediators as a potential explanation for TB-HIV related anemia [19–22]. For instance it has been reported that mild-to-moderate anemia is common during chronic inflammatory infections, including TB [21].

The percentage of anemia in PTB-HIV patients was 60% with the mean Hgb value of 10.8 ± 2.40 g/dl. This may be due to the influence of proinflammatory cytokines, such as IL-6 and TNF-a, which reduce production of erythropoietin, suppressed bone marrow response to erythropoietin, and altered iron metabolism, which may in turn impair erythropoiesis [23, 24].

This finding showed that Hgb level was significantly lower in PTB-HIV coinfected patients as compared to PTB patients and similar to a report in Addis Ababa, Ethiopia [25], Dar es Salaam, Tanzania [26], and Nigeria [3]. This might be due to a decreased erythrocyte life span, impaired marrow response, or impaired flow of iron from macrophages to the plasma in iron cycle metabolism. Decrease in hemoglobin also may be due to a nonimmune mechanism that develops secondary to granulomatous infiltration of the bone marrow [19, 21].

In this study, there was no significant difference in total WBC values between PTB and PTB-HIV coinfected patients. However, there were 6% and 14% leucopenia among PTB and PTB-HIV coinfected patients, respectively. These observed differences might be due to HIV coinfection. Leucopenia in HIV infection might be due to decrease in bone marrow production of granulocyte progenitor cells [27].

The mean value of neutrophils count of PTB patients was higher as compared to PTB-HIV coinfected patients in this study (4.6 ± 0.6 vs. 3.9 ± 0.5) with 22% and 66% prevalence of leucopenia, respectively. Since the study subjects included in this study were those with active tuberculosis, polymorphonuclear leukocytes (neutrophils) may be increased as part of the immune defense mechanism to defend the* Mycobacterium* infection. Neutropenia in PTB-HIV coinfected individuals may be due to consequences of the combined effect of hypersplenism and marrow granulopoietic failure mediated by different cytokines and malnutrition [28].

The other hematological parameter assessed in this study was platelet count. The result showed a statistically significantly higher platelet count in PTB patients when compared to PTB-HIV coinfected patients (336 ± 152 vs. 257 ± 132). This result is as the well-established expected value. The finding is supported by studies in Babylon province and Kirkuk city, Iraq [29, 30], and Pakistan [27]. These differences may be attributed to the reactive thrombocytosis which is found in a number of clinical situations including infectious diseases such as pulmonary tuberculosis due to increased thrombopoietic factors such as IL-6 which is released by inflamed cells as an inflammatory response [10, 24, 31]. The secretion of IL-6 in PTB patients will stimulate the production of platelets [32–34]. Some authors also reported the presence of autoantibody complexes as being responsible for the mild decreased platelet count in PTB-HIV infection [35].

The prevalence of thrombocytopenia among PTB patients was 8% while it was 20% among PTB-HIV coinfected patients. Different mechanisms such as immune mechanisms, bone marrow fibrosis, direct megakaryocyte infection, and hypersplenism had been implicated as possible causal factors for thrombocytopenia in PTB-HIV coinfected patients [36–40].

In the current study, we found high ESR values in both PTB and PTB-HIV coinfected patients (65 ± 36 mm/hr vs. 67 ± 29 mm/hr). However, there was no significant difference between the two groups. ESR value usually increased pulmonary tuberculosis [41]. Elevated ESR to a different level is one of the indicators of the severity of disease and as a prognostic tool. This might be due to alterations in the plasma proteins [42] which in turn affect ESR values.

In this study potential inflammatory related markers as the neutrophils extracellular nets (NETs), cytokines (IL-6, TNF-alpha, and IL-1), chemokines, iron, and vitamin D pathways disturbances related to TB were not assessed.

## 6. Conclusion

In this study, different hematological abnormalities in pulmonary tuberculosis with or without HIV are observed. There was high prevalence of neutropenia followed by anemia and thrombocytopenia. Hemoglobin, platelet, and neutrophils count values showed statistically significant difference between PTB and PTB-HIV coinfected patients. The WBC counts exhibited varying degree of alteration with high neutropenia and lymphopenia. A high ESR value was observed in all the study participants. Assessment of hematological parameters can be used as an indicator in the diagnosis and follow-up of PTB patients with or without HIV. We recommended assessment of PTB patients with or without HIV for various hematological disorders such as anemia, increased ESR, thrombocytosis, thrombocytopenia, and pancytopenia.

## Figures and Tables

**Table 1:** Sociodemographic characteristics of PPTB patients with and without HIV/AIDS at the University of Gondar Hospital, Gondar, Northwest Ethiopia.

**Characteristic**		**PTB **	**PTB-HIV**
		**N (**%**)**	**N (**%**)**
**Age in years **	15-24	16 (32)	9 (18)
	25-34	14 (28)	21 (42)
	35-44	11 (22)	13 (26)
	45-54	9 (18)	7 (14)

**Sex**	Female	25 (50)	24 (48)
	Male	25 (50)	26 (52)

**Resident**	Rural	31 (62)	20 (40)
	Urban	19 (38)	30 (60)

**Occupational status**	Housewife	10 (20)	10 (20)
	Merchant	3 (6)	4 (8)
	Farmer	20 (40)	5 (10)
	Government employee	2 (4)	7 (14)
	Daily laborer	7 (14)	16 (32)
	Student	8 (16)	8 (16)

**Table 2:** Comparison of hematological profiles of PTB patients with and without HIV.

**Hematological parameter**	**PTB **	**PTB-HIV**	***P* value**
	**Mean ± SD**	** Mean ± SD**	
WBC x 10^3^/ *µ*l	7.3 ± 3.1	6.9 ± 3.2	0.529
RBC x 10^6^/*µ*l	4.6 ± 0.85	4.12 ± 0.72	0 .108
Hgb (g/dl)	12.0 ± 3.4	10.8 ± 2.4	0.049*∗∗*
HCT (%)	38.3 ± 8.0	36.1 ± 6.1	0.114
MCV (fl)	91.7 ± 5	90.7 ± 4.3	0.056
MCH (pg)	29.3 ± 4.6	29.8 ± 5.5	0.612
MCHC (%)	34.2 ± 3.8	33.5 ± 3.2	0.282
PLT x 10^3^/*µ*l	336 ± 152	257 ± 132	0.007*∗∗*
Lymphocyte	1.8 ± 0.3	1.6 ± 0.4	0.273
Neutrophil	4.6 ± 0.6	3.9 ± 0.5	<0.001*∗∗*
Mixed cells	0.9 ± 0.4	1.4 ± 0.3	1.000
ESR (mm/hr)	65.3 ± 35.7	66.7 ± 28.6	0.827

**N.B:** *P value derived from independent t-test; ∗∗ indicates significant difference; mixed cells: monocyte, basophil, and eosinophil*.

## Data Availability

The data used to support the findings of this study are available from the corresponding author upon request.

## Abbreviations

AFB: Acid fast bacilli
BMI: Body mass index
CBC: Complete blood count
CD: Cluster of differentiation
EDTA: Ethylenediaminetetraacetic acid
ESR: Erythrocyte sedimentation rate
Hgb: Hemoglobin
HIV: Human immunodeficiency virus
ITP: Immune thrombocytopenic purpura
MCH: Mean cell hemoglobin
MCHC: Mean cell hemoglobin concentration
MCV: Mean corpuscular volume
PCV: Packed cell volume
PTB: Pulmonary tuberculosis
RBC: Red blood cell
TB: Tuberculosis
TLC: Total leukocyte count
WBC: White blood cell.
